# Special Issue “Advances in Biomedical and Tissue Engineering Strategies to Cross the Blood–Brain Barrier for Next-Generation Treatments Against Neurodegenerative Diseases”

**DOI:** 10.3390/ijms27010063

**Published:** 2025-12-20

**Authors:** Manuele Gori, Livia La Barbera

**Affiliations:** 1 Institute of Biochemistry and Cell Biology (IBBC)—National Research Council (CNR), International Campus “A. Buzzati-Traverso”, Monterotondo, 00015 Rome, Italy; 2 Molecular Neuroscience Unit, Università Campus Bio-Medico di Roma, 00128 Rome, Italy

## 1. Introduction

The blood–brain barrier (BBB) is a complex and semipermeable interface between the brain and systemic circulation, which is highly selective to the passage of blood cells, toxic components, nutrients, and metabolites into the central nervous system (CNS) [1]. As such, the BBB represents a major biological obstacle for delivering to the brain therapeutic amounts of drugs and molecules developed to treat neurodegenerative diseases and associated conditions [2,3]. Furthermore, being involved in preserving brain health and CNS homeostasis, BBB damage and disruption contribute to the onset and progression of neurological disorders by enabling the infiltration of harmful molecules and neurotoxic pathogens that trigger neuroinflammation [4,5,6]. Innovative strategies and improved drug delivery systems are therefore essential to bypass this natural shield and provide the CNS with targeted treatments and reliable diagnostics. Parallel efforts in bioengineering are equally crucial for developing advanced in vitro BBB models that more faithfully recapitulate the structure and function of this physiological barrier.

This Special Issue covers a set of 5 research articles and 2 reviews that explore the role played by the BBB in the context of neurological disorders. The present article collection provides an in-depth overview of the most recent tissue-engineering strategies to tackle neurodegenerative diseases, using groundbreaking solutions and pioneering biomedical approaches, ranging from micro- and nanoparticles to advanced in vitro models of the BBB, including microfluidic platforms and microphysiological systems.

In the first research article, Sampedro-Viana and coworkers highlight the importance of reliable in vivo models of chronic BBB dysfunction for dissecting the role of the BBB in neurological disorders in order to facilitate the effective delivery of drugs into the brain. The Authors developed an in vivo model of transient BBB opening, through an osmotic disruption mechanism, via periodic intravenous administration of mannitol over 12 weeks in healthy adult rats. This method, due to the induction of osmotic stress, caused a transient BBB opening and dysfunction (BBB leakage patterns). These phenomena were detected in a non-invasive manner using a sensitive contrast-enhanced magnetic resonance imaging (MRI)-based method. Furthermore, the Authors identified an association between osmotic BBB disruption and region-specific alterations in brain metabolism (i.e., specific metabolite signature) via real-time MR spectroscopy, as well as a link between chronic BBB dysfunction and neuroinflammation, by analyzing serum levels of pro-inflammatory cytokines (Contribution 1).

In the second research article, Lee and colleagues leveraged the potential of liposomes for the treatment of Alzheimer’s disease (AD) through the nose-to-brain delivery of three drugs (i.e., donezepil, memantine and BACE-1 siRNA) in a transgenic mouse model of AD. The intranasal administration of liposomes represents a non-invasive route to the brain, reducing off-target drug effects and minimizing their systemic adverse effects. The Authors demonstrated the individual and synergistic effects of the nose-to-brain-delivered liposome formulations on AD, and their therapeutic advantage compared with oral and intravenous administration in improving learning and memory abilities, as well as in reducing the brain accumulation of Aβ peptides and inflammatory cytokines in AD mice. These results provide an effective strategy to bypass the BBB for treating a range of neurodegenerative conditions, highlighting, at the same time, the potential of the proposed liposomal formulations for AD therapy (Contribution 2).

Advancing this technological perspective, Konig et al. introduce a compelling nanoparticle-based approach to BBB transport using transferrin-functionalized liposomes. Leveraging a sophisticated 3D microvascular network model (a BBB-on-a-chip), the Authors showed that transferrin-functionalized liposomes markedly improve the translocation of the Microtubule-Associated Protein Tau (MAPT)-targeting antisense oligonucleotides—an approach with strong therapeutic promise for tauopathies and related neurodegenerative diseases. Their work highlights the power of combining biomimetic 3D BBB platforms with ligand-directed nanocarriers to achieve targeted, efficient, and non-disruptive delivery of tau-targeting antisense oligonucleotides to the CSN. This study exemplifies the next generation of bioengineered strategies designed to overcome the BBB and accelerate the development of precision treatments against neurodegeneration (Contribution 3).

In the fourth research contribution, Guillot and coworkers showed the useful role played by other extracellular nanovesicles, the exosomes from sera of elderly patients with severe obstructive sleep apnea syndrome (OSAS), as novel potential biomarkers of OSAS and associated cognitive disorders, using an in vitro model of the BBB. The Authors reported elevated levels of serum exosomes containing Aβ and tau proteins in the brain of elderly OSAS patients compared to controls, along with increased BBB permeability, thereby hypothesizing a role for the exosomes as potential biomarkers of neurocognitive risks, with possible involvement in the development of neurodegenerative diseases. The present study, by establishing a link between severe OSAS and BBB dysfunction in the elderly, identifies serum exosomes as an early and minimally invasive tool for the diagnosis of OSAS in neurocognitive diseases, also spurring the search for novel therapeutic strategies (Contribution 4).

As a part of the Special Issue, the review by Nuzzo et al. examines the emerging role of natural extracellular vesicles (EVs), plant-derived extracellular vesicles (PEVs) and artificial vesicles (AVs) in neurodegenerative diseases, as both biomarkers and therapeutic tools, focusing on their capacity to traverse the BBB and deliver therapeutics in a safe and efficient manner. Here, the Authors summarize the multiple advantages provided by nanobiotechnologies, using EVs, including exosomes and microvesicles, as well as AVs, for selectively targeting specific brain regions and cell types, thereby increasing the efficacy of diagnosis and therapies against several brain disorders. Nuzzo and colleagues describe the therapeutic mechanisms of action of the different vesicles and their effects in various neural disorders, from AD to Parkinson’s disease (PD) and multiple sclerosis (MS). These nanotools offer unique opportunities in the field of nanomedicine for targeted and controlled drug delivery with reduced potential adverse effects, enabling them to bypass the BBB and improving drug stability and solubility. Hence, nanovesicles seem to hold great promise for the development of new diagnostic and therapeutic approaches in the treatment of neurodegenerative diseases (Contribution 5).

In the next research work, Mokhtari et al. presented important clinical insights into the management of severe neurotoxicity associated with Chimeric Antigen Receptor T-cell (CAR-T) therapy, which represents a growing medical challenge as cellular immunotherapies expand into the field of neuro-oncology. The Authors evaluated the use of intravenous immunoglobulin (IVIG) as a supportive strategy to mitigate neuroinflammatory complications, highlighting how targeted immunomodulation may help preserve neurological function without diminishing therapeutic efficacy. This work reinforces the need for finding innovative methods capable of stabilizing or protecting the CNS through approaches that align closely with emerging biomedical and tissue engineering strategies aimed at overcoming the BBB obstacle, enabling safer, next-generation treatments for neurodegenerative and neuroinflammatory conditions (Contribution 6).

Finally, the review by Ferrari et al. offers a forward-looking perspective on the role of microfluidic-based technologies in overcoming the significant challenges of the BBB transport in neurodegenerative diseases, such as AD. By integrating advanced lab-on-a-chip platforms with disease-relevant cellular models and the microfluidic generation of functionalized nanocarriers for targeted drug delivery, the work highlights how microfluidics can simulate BBB dysfunction and enable high-throughput drug testing, through the synthesis of more precise drug delivery systems, to accelerate the development of therapeutics tailored for neurodegenerative disorders. The Authors also emphasize the remaining translational barriers—scalability, physiological complexity, process reproducibility and integration with emerging micro- and nanomaterial-based technologies—making their review an up-to-date roadmap for researchers seeking innovative tissue engineering and nanomedicine strategies to penetrate the BBB and address the unmet clinical needs in AD and other neurodegenerative diseases (Contribution 7).

## 2. Conclusions

The articles published in this Special Issue, “Advances in Biomedical and Tissue Engineering Strategies to Cross the Blood–Brain Barrier for Next-Generation Treatments against Neurodegenerative Diseases”, represent an overview of the latest research on the prevention and treatment of neurodegenerative diseases, placing the BBB at the center of the study, thereby exploring some state-of-the-art methods and innovative technologies aimed at overcoming this biological barrier to deliver effective diagnostics and care to the CNS. Particular emphasis is given to the use of nanoparticles (ranging from liposome-based drug carriers to natural and synthetic extracellular vesicles) as versatile vehicles for crossing the BBB to target the CNS in the treatment of AD and other neurodegenerative disorders, including exosomes as promising diagnostic tools. Altogether, these contributions underline also the interesting ability of circulating EVs in communicating between different tissues and body districts, throughout the whole organism, with multiple useful diagnostic and therapeutic implications, as well as prospective beneficial outcomes for disease interventions. In some of the research works collected herein, the Authors developed novel biomedical approaches using different technologies for investigating the neurotoxicity and neuroinflammation associated with BBB breakdown by harnessing in vivo and alternative 3D in vitro models of damaged BBB to study its involvement in neurological disorders. The two highly informative review articles included in this Special Issue provide an extensive overview of the most recent nanomedicine- and microfluidic-based technologies, not only for the development of advanced models of the BBB-on-a-chip, but also for the synthesis of nano- and micro-particle drug delivery systems, including their therapeutic mechanisms of action for clinical applications in neurodegenerative diseases. Collectively, all these contributions, encompassing a broad spectrum of medical and experimental subjects, deepen our understanding of the BBB function in both physiological conditions and brain disorders, shedding new light on and advancing our knowledge of the roles played by this natural protective membrane, especially in the framework of neurodegenerative diseases.
